# Proactive Control: Neural Oscillatory Correlates of Conflict Anticipation and Response Slowing

**DOI:** 10.1523/ENEURO.0061-17.2017

**Published:** 2017-05-26

**Authors:** Andrew Chang, Jaime S. Ide, Hsin-Hung Li, Chien-Chung Chen, Chiang-Shan R. Li

**Affiliations:** 1 Department of Psychology, National Taiwan University, Taipei, Taiwan 10617; 2 Department of Psychiatry, Yale University, New Haven, CT 06520; 3 Center for Neurobiology and Cognitive Science, National Taiwan University, Taipei, Taiwan 10617; 4 Department of Neuroscience, Yale University, New Haven, CT 06520; 5 Interdepartmental Neuroscience Program, Yale University, New Haven, CT 06520; 6 Beijing Huilongguan Hospital, Beijing 100096, China

**Keywords:** Bayesian model, Electroencephalogram (EEG), neural oscillation, proactive control, stop-signal task

## Abstract

Proactive control allows us to anticipate environmental changes and adjust behavioral strategy. In the laboratory, investigators have used a number of different behavioral paradigms, including the stop-signal task (SST), to examine the neural processes of proactive control. Previous functional MRI studies of the SST have demonstrated regional responses to conflict anticipation—the likelihood of a stop signal or P(stop) as estimated by a Bayesian model—and reaction time (RT) slowing and how these responses are interrelated. Here, in an electrophysiological study, we investigated the time–frequency domain substrates of proactive control. The results showed that conflict anticipation as indexed by P(stop) was positively correlated with the power in low-theta band (3–5 Hz) in the fixation (trial onset)-locked interval, and go-RT was negatively correlated with the power in delta-theta band (2–8 Hz) in the go-locked interval. Stimulus prediction error was positively correlated with the power in the low-beta band (12–22 Hz) in the stop-locked interval. Further, the power of the P(stop) and go-RT clusters was negatively correlated, providing a mechanism relating conflict anticipation to RT slowing in the SST. Source reconstruction with beamformer localized these time–frequency activities close to brain regions as revealed by functional MRI in earlier work. These are the novel results to show oscillatory electrophysiological substrates in support of trial-by-trial behavioral adjustment for proactive control.

## Significance Statement

Proactive control is central to adaptive behavior. Many functional MRI studies have dissected the neural basis of conflict processing and behavioral adjustment, but evidence from electrophysiology is fragmentary. Here, by combining EEG and a stop-signal task, we demonstrated distinct frequency domain substrates of conflict anticipation, RT slowing, and stimulus prediction error. In particular, neural activities of conflict anticipation preceded RT slowing, and the power of these activities were correlated, in support of proactive control of behavior. Further, beamformer analysis localized the sources of these activities as revealed by functional MRI. These new findings complement the literature by specifying the electrophysiological correlates of trial-by-trial response control within a single paradigm.

## Introduction

The ability to make plans and proactively adjust our behavior is critical to survival. Proactive control has been studied with many different behavioral paradigms in which participants are cued to upcoming events (Vink et al. 2005; Brass and Haggard, 2007, Chikazoe et al. 2009; Kühn et al. 2009; Jahfari et al. 2010; Horga et al. 2011; Leunissen et al. 2016). In the stop-signal task (SST), for instance, frequent motor responses are to be interrupted occasionally by a stop signal, and increased stop signal probability bolsters proactive control, as shown in slower response time and delayed motor cortical activity (Jahfari et al. 2010). Participants typically demonstrate slower reaction time (RT) after a stop signal, or postsignal slowing (PSS), in the SST. As investigated extensively in behavioral studies, PSS does not reflect the surprise effects of stop signal but rather a global, contextual modulation of signal expectancy on motor response (Morein-Zamir et al. 2007; Bissett and Logan, 2011). In diffusion models of SST, response thresholds increased when stop signals were expected to occur, and participants made response-strategy adjustments on a trial-by-trial basis, suggesting proactive adjustment to changing environments (Verbruggen and Logan, 2009b). On the other hand, the neural mechanisms underlying trial-by-trial behavioral adjustment have been examined only recently under a formal computational framework.

In a previous functional MRI (fMRI) study of the SST, authors applied a dynamic Bayesian model to estimate trial-by-trial probability of the stop signal—P(stop)—and showed that higher P(stop) is associated with prolonged go trial RT, indicating proactive control of motor response (Hu et al. 2015a). The gist of the study was to identify and distinguish regional brain activations specific to signal anticipation and response slowing as a result of the anticipation. In modeling fMRI signals at trial and target onsets, they showed that the anterior presupplementary motor area (pre-SMA) responds to increased P(stop) in correlation with activations of the posterior pre-SMA and bilateral anterior insula during prolonged RT. These findings associate conflict anticipation with its motor consequence.

Many studies identified time-domain event-related potentials (ERPs) in association with proactive control, including processes related to switch preparation or anticipatory attention (Karayanidis and Jamadar, 2014; Angelini et al. 2016; Di Russo et al. 2016; Langford et al. 2016). Neural oscillations represent frequency-domain electrophysiological excitabilities of neuronal populations, with oscillations in different frequencies reflecting dynamic information exchange between distinct functional networks (Fries, 2005). Electroencephalogram (EEG) studies have shown that neural oscillations measured above the medial-frontal cortex are associated with cognitive control (Cavanagh and Frank, 2014; Cohen, 2014a). In Simon tasks, the power in theta band (∼3–8 Hz) increased and positively predicted RT in high conflict trials (Pastötter et al. 2010, 2013; Cohen and Cavanagh, 2011; Nigbur et al. 2011; Cohen and Donner, 2013; Cohen and Ridderinkhof, 2013; Clayton et al. 2015). Proactive and reactive controls were each associated with theta oscillations originating from distinct cortical networks during task switching (Cooper et al. 2015). In contrast, delta band (∼1–3 Hz) power was associated with shorter RT during error trials in the Simon task (Cohen and van Gaal, 2014). Proactive versus reactive control modulated the power of delta oscillations differently in an SST (Lavallee et al. 2014). Alpha (∼8–13 Hz) power, localized in the superior frontal cortex, was associated with attentional control to inhibit irrelevant sensory inputs in a flanker task (Suzuki and Shinoda, 2015). In a magnetoencephalography study of eye movement control, alpha to low beta bands (∼10–18 Hz) increased in power in the frontal eye field during suppression of a prepotent saccade compared with generation of an automatic saccade, an effect that positively predicted performance accuracy (Hwang et al. 2016). Together, these studies characterized oscillatory correlates of various forms of proactive control, but the frequency domain processes of conflict anticipation and response control remain to be dissociated and related to behavioral performance.

To fill this gap of research, we used a dynamic Bayesian model (Yu and Cohen, 2009) to quantify the extent of conflict anticipation—belief of an upcoming stop signal—trial by trial, on the basis of trial history. To investigate the neural oscillatory basis of proactive control, we identified the EEG time–frequency correlates of conflict anticipation and RT slowing in trial-by-trial analyses and used beamforming methods to localize the sources of these correlates. Specifically, we hypothesized that the time–frequency correlates of conflict anticipation should precede in time and correlate in power with those of RT slowing.

## Methods

### Participants

Eighteen healthy adults (9 males, 22.6 ± 1.3 years of age), who were all students and naive to the purposes of the experiment, participated in the study. All provided written consent and were financially compensated for their participation, in accordance with the guideline of Helsinki Declaration and a protocol approved by the Research Ethics Committee of National Taiwan University.

### Behavioral task

We used a simple RT task of the stop signal paradigm (Chang et al., 2014; Chang et al., 2015; Li et al., 2008; Verbruggen and Logan, 2009a). There were two trial types, “go” and “stop,” randomly intermixed in presentation with a ratio of ∼3:1. The intertrial interval was 2 s. A small white dot (fixation) appeared at the center of a black screen to engage attention at the beginning of every trial. After an interval ranging randomly from 1 to 3 s (the foreperiod), the dot turned into a circle (∼2° of visual angle), which served as a go signal. The participants were instructed to quickly press a button at go-signal onset but not before. The circle vanished either at button press or 1 s after go-signal onset, whichever came first, and the trial terminated. A premature button press before go-signal onset also terminated the trial. In the stop trial, an additional X, the stop signal, replaced the go signal. The participants were instructed to withhold button press on seeing the stop signal. The trial terminated at the button press or 1 s after stop-signal onset. The duration between go- and stop-signal onsets, or the stop-signal delay (SSD), was determined by a staircase procedure. The one-up-one-down procedure (Levitt, 1971) started with an SSD of 200 ms, and increased and decreased by 64 ms each after a successful and failed stop trial. By increasing and decreasing each SSD after a stop success and error, the staircase procedure allows participants to succeed in approximately half of the stop trials.

The task was divided into four sessions, each with 100 trials and lasting no longer than 8 min, with a short break in between sessions. There were ∼5 min of practice on the task before the experiments to ensure that participants understood the task. Participants were instructed to “respond to the go signal quickly while watching out for the stop signal, which might appear occasionally.”

### Bayesian modeling of the sequential effect

We used a dynamic Bayesian model (Yu and Cohen, 2009) to estimate the belief of an upcoming stop signal on each trial, P(stop), based on preceding stimulus history. In the model, previous trial information is encoded in the prior distribution. In every trial, the posterior distribution is carried over to the next trial as a prior. Although the stop and go trials were randomized in presentation, participants learned from local trial structure and updated their belief of P(stop) and adjusted RT on a trial-by-trial basis. P(stop) reflects participants’ estimation of the trial-by-trial likelihood of the stop signal on the basis of the Bayesian model, and it is computed independent of the RT. A sequential effect is defined as the linear correlation between P(stop) and RT for all go trials (Ide et al. 2015).

In the model, participants have the knowledge that each trial has a probability *r_k_* for being a stop trial and 1−*r_k_* for being a go trial. Furthermore, the probability *r_k_* has a chance α to be the same as *r_k–1_* and 1 – α being resampled from a prior distribution π(*r_k_*).

With these generative assumptions, participants use Bayesian inference to update their prior belief of seeing a stop signal on trial *k*, $p\left(r_{k}|s_{k-1}\right)$ based on the posterior of the last trial $p\left(r_{k-1}|s_{k-1}\right)$ given the last trial’s true category (**s***_k_* = 1 for stop trial, *s_k_* = 0 for go trial), where ***s**_k_* = {*s_1_*, …, *s_k_*} is shorthand for all trials 1 through *k*. Specifically, given that the posterior distribution was $p\left(r_{k-1}|s_{k-1}\right)$ on trial *k* − 1, the prior distribution of stop signal in trial *k* is given by

$$p\left(r_{k}|s_{k-1}\right)=\alpha *p\left(r_{k-1}|s_{k-1}\right)+\left(1-\alpha \right)*\pi \left(r_{k}\right),$$

where the prior distribution *π*(*r_k_*) is a beta distribution with prior mean *pm* and shape parameter *sc*, which were reparameterized from the *beta*(*a*,*b*) distribution such that *pm* = *a*/(*a* + *b*) and *sc* = (*a* + *b*), and the posterior distribution is computed from the prior distribution and the outcome according to Bayes’ rule:

$$p\left(r_{k}|s_{k}\right)\propto P\left(s_{k}|r_{k}\right)*p\left(r_{k}|s_{k-1}\right).$$

The Bayesian estimate of the probability of trial *k* being a stop trial—which we colloquially call P(stop)—given the predictive distribution $p\left(r_{k}|s_{k-1}\right)$ is expressed by

$$P\left(s_{k}=1|s_{k-1}\right)=\int P\left(s_{k}=1|r_{k}\right)*P\left(r_{k}|s_{k-1}\right)dr_{k}=\int r_{k}*P\left(r_{k}|s_{k-1}\right)dr_{k}=\;〈r_{k}|s_{k-1}〉.$$

We approximated the mean of the predictive distribution $p\left(r_{k}|s_{k-1}\right)$ by the maximum a posteriori (MAP) estimate ${\hat{r}}_{k}=\arg\underset{r_{k}}{\max}p\left(r_{k}|s_{k-1}\right)$. The proposition that the predictive distribution is a mixture of the previous posterior distributions and a generic prior distribution is essentially equivalent to using a causal, exponential, linear filter to estimate the current rate of stop trials (Yu and Cohen, 2009). In summary, for each participant, given a sequence of observed go/stop trials and the three model parameters {α, *pm*, *sc*}, we estimated P(stop) for each trial. Generally speaking, α quantifies the weight given by the participant to the previous trials (the magnitude of influence from previous to current trial), *pm* is the mean of the fixed belief of stop signal, and *sc* reflects how skewed the distribution is around the mean. It is worth noting that the Markov modeling approach (transition between the static and dynamic states) is assumed to be of the first order.

To obtain the best fit parameters for sequential effect in each individual, we grid-searched for the parameters that maximized the Pearson correlation between go-RT and P(stop) using Matlab. The search space of model parameters were set to the following ranges: α = [0.02, 0.04, …, 0.98], *pm* = [0.01, 0.03, …, 0.49], and fixed *sc* = 10. For the stop trials, we also quantified the prediction error (PE), or Bayesian surprise, as |1 – P(stop)| (Ide et al. 2013).

### Electroencephalography acquisition and data preprocessing

The EEG was collected with a whole-head, 256-channel geodesic EEG system with HydroCell Sensor Nets (Electrical Geodesics). This system provides uniform spatial sampling (∼2 cm, sensor to sensor), covering the entire scalp surface and extending 120° in all directions from the vertex reference electrode. The EEG was amplified at a gain of 1000 and recorded with a vertex physical reference. Signals were digitized at 500 Hz with a 16-bit analog-to-digital converter, which allowed an amplitude resolution of 0.076 μV. The computer administering the task sent a digital trigger to the recording system at the onset of fixation and go signal of every trial.

The data were preprocessed by the following procedures offline before statistical analysis with the Brain Electrical Source Analysis (BESA) software package. First, the raw data were filtered by a 0.7- to 100-Hz bandpass (FIR) filter. Second, independent component analysis (ICA) decomposition using an extended infomax algorithm (Lee et al. 1999) was performed on the continuous EEG recording of each session for each participant, and the channel-time EEG data were projected into the component-time EEG data in the ICA space, with PCA dimension reduction excluding the independent components with <0.75% of variance. ICA is a useful technique for artifact removal on EEG recordings by spatially separating independent artifact sources mixed with the brain activities of interest in the surface EEG channels. Third, the components reflecting artifact (identified by visual inspection), including eye blinking, eye movement, electrocardiogram, and 60-Hz powerline noise, were excluded. Fourth, the data of the remaining components were projected back as ICA artifact-removed 256-channel EEG data. Fifth, to identify any remaining artifacts that were not removed by ICA, the ICA artifact-removed data were epoched from –500 to 1000 ms, each time-locked to the onset time of fixation signal or go signal. In the final step, the epochs with voltage change exceeding 150 μV at any channel were identified as containing artifact and excluded for further analyses. As a result of the preprocessing, 85.9 ± 15.0% of the trials were included for further analyses.

### Time–frequency decomposition

We examined EEG activities across time and frequencies, which may reflect the activities of different neuronal populations in association with distinct cognitive functions. The Morlet wavelet transformation was computed for each time point continuous on the ICA artifact-removed 256-channel unfiltered EEG data with 59 logarithmically spaced frequency bins between 2 and 50 Hz. The wavelet was designed such that the half-maximum width was equal to 1 period of the lowest frequency with the width equal to 4 periods of the highest frequency, linearly interpolated for each frequency bin in between. Thus, EEG data were transformed into power at each time–frequency bin. Subsequently, we segmented the time–frequency data from –500 to 1000 ms into epochs, time-locked to the onset of fixation signal, go signal, or stop signal, and the power of each epoch was baseline corrected to the mean power of the –300- to –100-ms period for each frequency bin. We performed the time–frequency decomposition before epoching the data, circumventing the issue of edge artifact as can occur in time–frequency decomposition on short EEG epochs. There was a buffer zone of at least 4 s at the beginning and the end of each continuous EEG recording.

### Correlating time–frequency power with sequential effect

We performed a Spearman rank correlation on the power for each time–frequency bin with P(stop), go-RT, or PE on the fixation (trial onset)-locked, go-locked, and stop-locked interval across trials for each participant. To examine within-subject correlations on P(stop) and EEG across all participants, we used the group-averaged parameters {α, *pm*, *sc*} of individual Bayesian model that maximized the sequential effect for each participant, according to previous model-based studies (Camerer and Ho, 1999; O’Doherty et al. 2004; Ide et al. 2015). Further, we focused on the surface channel FCz (standard channel montage from BESA), located at midfrontal areas, because previous EEG studies of conflict processing reported activities mainly from the medial frontal cortex (Cavanagh and Frank, 2014; Cohen, 2014a) and fMRI studies described activations to conflict anticipation, RT slowing, and prediction error in distinct areas of the medial prefrontal cortex (Hu et al. 2015a).

To control for type I error of multiple comparisons, we performed a nonparametric cluster-based permutation test (Cohen, 2014b; Maris and Oostenveld, 2007) on the 2D time–frequency maps in two loops. In the first loop, within each participant, we randomly permuted trial mapping between EEG data and behavioral responses for 1000 times and computed EEG–behavior correlation coefficient for each permutation as a null distribution for each time–frequency bin. We computed the *z*-value of the observed correlation coefficient relative to the null distribution for each time–frequency bin—a *z*-map—to represent the EEG–behavior correlation for each participant (Cohen, 2014b).

In the second loop, we tested the random effect hypothesis of specific time–frequency bins showing consistent EEG–behavior correlation across participants. First, we used a two-tailed one-sample *t* test to examine whether any time–frequency bins on the *z*-map were significantly different from 0 across participants. Second, we grouped adjacent time–frequency bins reaching a threshold of *p* < 0.02, 0.005, or 0.001 into single clusters and summed the *t*-value within each cluster into a cluster-level statistic. Third, we built a permutation null distribution with 2000 iterations from the observed *z*-maps, representing zero correlation across participants. Multiple comparisons were controlled by extracting the summed *t*-value of the largest suprathreshold cluster of each iteration into null distribution, and the final *p*-value (*p*_perm_) was computed by ranking the clustered *t*-value in the real data relative to the null distribution (Maris and Oostenveld, 2007), where the 2000 iterations of permutation divided the *p*_perm_ with a step size of 1/2000, ranging from 0 to 1. The observed statistical power was estimated by repeating the permutation test 200 times and then taking the percentage of obtaining *p*_perm_ < 0.05 out of the 200 tests.

### Beamformer source reconstruction

We used beamformer to estimate the source location of the time–frequency activities of interest. As a signal-processing technique to discriminate between signals arriving from a location of interest and signals arriving from other locations, beamformer is used to spatially filter scalp-recorded EEG data to estimate the source power for a specific location in the brain. Specifically, by considering signals generated from one specific voxel while attenuating signals from other voxels, the distribution of source power of the 3D brain is estimated by repeatedly constructing beamformer at each voxel (Green and McDonald, 2009). We used the multiple source beamformer algorithms in BESA (Gross et al. 2001) to estimate the source of the time–frequency cluster at the single-trial level for non–phase-locked time–frequency activities. For each voxel, the source power in the time–frequency cluster of interest *P* is normalized by the source power in a reference time–frequency cluster *P_ref_* as *q*:

$$\begin{array}{l} q=\sqrt{P/P_{ref}}-1,\;\text{for}\;P\geq P_{ref};\text{ or}\\ q=1-\sqrt{P_{ref}/P},\;\text{for}\;P<P_{ref}. \end{array}$$

For a specific voxel, the magnitude of positive *q* represents the magnitude of *P* ≥ *P_ref_*, whereas the magnitude of negative *q* represents the magnitude of *P* < *P_ref_*.

We estimated a *q*-map for each individual cluster for each participant. To reconstruct the sources of each time–frequency cluster associated with the sequential effect, we median-split the trials based on P(stop) or go-RT for each participant. We did not reconstruct the source of PE owing to an insufficient number of stop trials available for analysis (*n* ≤ 100). We assigned beamformer power source of the time–frequency cluster, each in the higher and lower half of the behavioral index, as *P* and *P_ref_*, respectively. That is, the voxels with largest positive and largest negative *q* values are likely the source of the time–frequency cluster correlates each of P(stop) and go-RT, respectively.

We used the four-shell ellipsoidal model implemented in BESA toolbox to solve the inverse problem. Although our head model was not MRI-based and represented a simplistic approximation to the realistic brain, empirical simulations showed that the four-shell head model localizes the source with ∼1 cm of averaged errors (Cohen et al. 1990; Cuffin et al. 1991), which is reasonably accurate (Slotnick, 2004). The locations of all 256 channels were provided by BESA for the 256-channel HydroCel Geodesic Sensor Net.

To perform source localization at the group level, we coregistered and normalized individual *q*-maps with affine transformation to reduce misalignment among the images, and performed one-sample *t* tests. These analyses were performed with Statistical Parametric Mapping (SPM12, Wellcome Department of Imaging Neuroscience, University College London, UK).

## Results

### Behavioral performance and sequential effect

The go success rate was 98.4 ± 1.9% (mean ± SD), and go-RT was 405 ± 64 ms across participants. The stop success rate was 48.7 ± 2.2%, suggesting the success of the staircase procedure in tracking participants’ performance and eliciting errors in approximately half of the stop trials. The RT of stop-error trials was 360 ± 52 ms, which was significantly shorter than go-RT (*t*_(17)_ = 10.81, *p* < 0.001^a^). The critical SSD was computed by a maximal likelihood procedure on the sequence of all staircase-generated SSDs for each participant, and the stop-signal reaction time (SSRT) was computed by subtracting the critical SSD from the median go-RT for each participant, based on the race model. The critical SSD was 170 ± 71 ms, and SSRT was 216 ± 25 ms. These behavioral outcome were within the range reported in earlier studies (e.g., Logan et al. 1997; Li et al. 2008; Verbruggen and Logan, 2008; Ide et al. 2013; Hu et al. 2015a; Schevernels et al. 2015; Langford et al. 2016).

**Figure 1.**
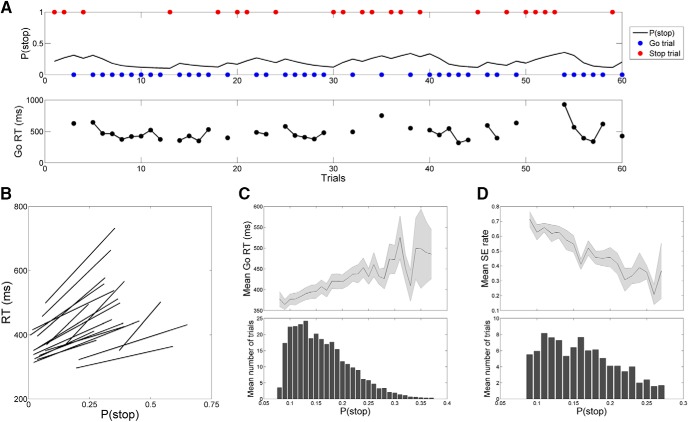
Bayesian model prediction of behavioral performance in the stop-signal task. ***A***, Example sequence of trials. The upper panel shows the sequence of go (blue dots) or stop (red dots) trials and how Bayesian belief about encountering a stop trial [P(stop), black line] increases and decreases, respectively, after each stop and go trial. The lower panel shows the sequence of go-RT in the upper panel. Overall, go-RT tended to be prolonged with a higher P(stop). ***B***, Correlation between P(stop) and RT across all go success trials, with each regression line representing an individual participant. ***C***, Positive correlation between go-RT and P(stop) collapsed over all participants. The plot in the upper panel shows the mean ± SE, the histogram in the lower panel shows the distribution of P(stop), and both were binned at intervals of 0.01. ***D***, Negative correlation between stop error (SE) rate and P(stop), with the same format as in ***C***.

We fitted a dynamic Bayesian model for the trial sequence of each participant (Fig. 1*A*), and the best-fitted parameter values, with *sc* fixed at 10, were α = 0.78 ± 0.14 (mean ± SD) and *pm* = 0.14 ± 0.16 across participants. The Pearson correlation coefficient between P(stop) and go-RT was 0.29 ± 0.08 (range, 0.14–0.43; Fig. 1*B*), with *p* < 0.001 for 16 participants and *p* = 0.005 and 0.016 for the other 2 participants. At the group level, we first estimated the P(stop) for each participant, took the average of go-RT and stop error (SE) rate of the trials at each P(stop) bin (width = 0.01) within each participant, and then averaged the mean go-RT or mean SE rate across participant on each bin. We truncated the extreme P(stop) bins in which very few participants (*n* ≤ 2) were represented, and the resulting P(stop) bins were each in the range of [0.08, 0.37] and [0.09, 0.27] for correlation with grand-averaged go-RT and SE rate. Pearson correlation showed a positive correlation between P(stop) and the grand-averaged go-RT (*r*_(29)_ = 0.89, 95% CI = [0.77, 0.94], *p* < 10^−10b^; Fig. 1*C*) and a negative correlation between P(stop) and grand-averaged SE rate (*r*_(18)_ = –0.93, 95% CI = [–0.82, –0.97], *p* < 10^−8c^; Fig. 1D). Together, the results showed that the dynamic Bayesian model successfully captured the sequential effect for each participant as well as for the group.

### Neural time–frequency power correlates

To examine for which frequency bands and at what time interval the time–frequency activities were related to sequential effects, we correlated the time–frequency power at FCz channel in midfrontal region with P(stop) and go-RT each in fixation-locked and go-locked epochs across trials within each participant. As described in detail earlier, we evaluated the statistical significance of the correlations across participants in random effects analysis on the basis of a cluster-based nonparametric permutation test.

**Figure 2.**
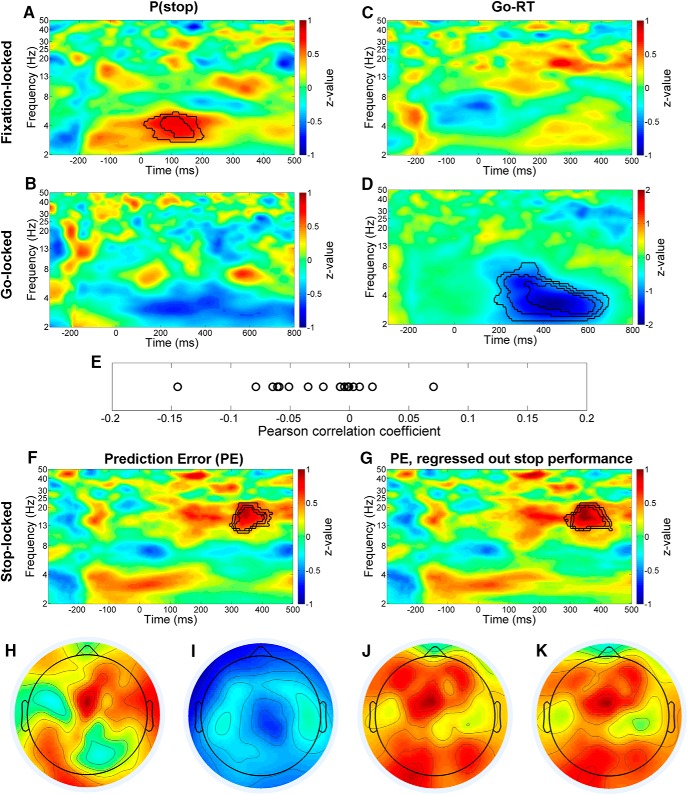
Trial-by-trial oscillatory power correlates of P(stop), go-RT, and PE at channel FCz. ***A***, Correlation between fixation-locked (onset at 0 ms) power and P(stop) at each time–frequency bin. The color represents the *z*-value of Spearman correlation, and the black contours represent statistically significant time–frequency clusters in the nonparametric cluster-based permutation test across participants, with clustering threshold at *p* < 0.02, 0.005, and 0.001 levels (see Materials and Methods for details). It showed a positive correlation in the intervals 3–5 Hz and 0–200 ms. ***B***, No clusters showed a significant correlation between go-locked power and P(stop). ***C***, No clusters showed a significant correlation between fixation-locked power and go-RT. ***D***, Correlation between go-locked power and go-RT, with the permutation test showing a negative correlation in the intervals 2–8 Hz and 200–700 ms. ***E***, The coefficient of trial-by-trial Pearson correlation between the mean power of the clusters identified in ***A*** and ***D*** of individual participants. Wilcoxon signed rank test showed that the correlation coefficients across participants were significantly below zero. ***F***, ***G***, Correlation between stop-locked power and PE, with the permutation test showing a positive correlation in the intervals 12–22 Hz and 300–400 ms. The topographies of each correlational cluster (*p* < 0.005) are shown in the bottom panel, where ***H***, ***I***, ***J***, and ***K*** each show the cluster of ***A***, ***D***, ***F***, and ***G***, all with the strongest correlations at the midfrontal region. We performed the same analyses at Pz channel (Fig. 2-1).

In the fixation-locked epoch, permutation test on the 0-500 ms range showed that P(stop) was positively correlated with the power in the cluster at 3-5 Hz (low-theta band) and 0-200 ms interval (*p*_perm_ = 0.034^d^, cluster threshold at *p* < 0.02), with the strongest correlation at ∼4–5 Hz and 100–150 ms (*p*_perm_ = 0.036^e^, cluster threshold at *p* < 0.005; Fig. 2*A*). In contrast, the go-RT was not correlated with power in this time–frequency range (Fig. 2*B*).

10.1523/ENEURO.0061-17.2017.f2-1 Figure 2-1 Trial-by-trial oscillatory power correlates of P(stop), go-RT, and PE at channel Pz. The format is the same as with Fig. 2. The color represents the *z*-value of Spearman correlation, and the black contours represent statistically significant time–frequency clusters in the nonparametric cluster-based permutation test across participants, with clustering threshold at *p* < 0.02, 0.005, and 0.001 levels (see Materials and Methods for details). ***A***, ***B***, Time–frequency power was not correlated with P(stop) in fixation-locked or go-locked epoch. ***C***, Time–frequency power was not correlated with go-RT in fixation-locked epoch. ***D***, Time–frequency power was negatively correlated with go-RT in the intervals 2–8 Hz and ∼100–500 ms in go-locked epoch. ***E***, ***F***, Time–frequency power was not correlated with PE in stop-locked epoch.. Download Figure 2-1, TIF file.

In the go-locked epoch, permutation test showed that P(stop) was not correlated with the power over the 0-800 ms range (Fig. 2*C*). In contrast, go-RT was negatively correlated with power at 2–8 Hz (across delta and theta bands) in the 200-700 ms interval (*p*_perm_ = 0.005^f^, cluster threshold at *p* < 0.02), with the strongest correlation at 2.5–4.5 Hz and 300–600 ms (*p*_perm_ = 0.002^g^, cluster threshold at *p* < 0.001; Fig. 2*D*).

Given that P(stop) and go-RT were correlated as sequential effects (Fig. 1*B*, *C*), we further tested whether the power of the time–frequency clusters each associated with P(stop) and go-RT was correlated across trials within each participant. For each participant, we correlated the mean power of P(Stop) and go-RT clusters (with cluster threshold at *p* < 0.005 and 0.001, respectively) in a Pearson regression. Because the distribution of the correlation coefficients violated normality assumption (Kolmogorov–Smirnov test: *p* < 0.001), we used a two-tailed Wilcoxon signed-rank test to examine the group result. The test showed that the correlation coefficients were significantly below 0 (i.e., negative correlation) across all participants (exact *p* = 0.024^h^, 95% CI = [–0.047, –0.002]; Fig. 2*E*).

We also correlated the power at FCz channel with PE in stop-locked epoch across trials. In the stop-locked epoch, the nonparametric permutation test in the 0-500 ms range showed that PE was positively correlated with the power in the cluster at 12–22 Hz (low-beta band) and 300–400 ms (*p*_perm_ = 0.021^i^, cluster threshold at *p* < 0.02), with the strongest correlation at ∼13–18 Hz and 320–380 ms (*p*_perm_ = 0.004^j^, cluster threshold at *p* < 0.001; Fig. 2*F*). We further considered the possibility that this correlation was confounded with stop trial performance, given that P(stop) was associated with SE rate (Fig. 1*D*). Thus, we regressed out the mean power of stop-success and stop-error trial and performed the correlation of the residual power with PE across all stop trials. The result remained that the PE was positively correlated with the power in the cluster at 12–22 Hz and 300–400 ms (*p*_perm_ = 0.020^k^, cluster threshold at *p* < 0.001), with the strongest correlation at ∼13–19 Hz and 310–400 ms (*p*_perm_ = 0.001^l^; Fig. 2*G*).

We performed additional analyses on the time–frequency power at Pz channel in parietal-midline region (Fig. 2-1), following the same procedures on FCz. The P(stop) was not correlated with time–frequency power in either fixation-locked or go-locked epoch (Fig. 2-1*A*, *B*). The go-RT was not correlated with time–frequency power in fixation-locked epoch (Fig. 2-1*C*). However, the go-RT was negatively correlated with the power in the cluster at 2–8 Hz (across delta and theta bands) and ∼100–500 ms (*p*_perm_ < 0.001^m,n,o^, cluster threshold at *p* < 0.02, 0.005, or 0.001; Fig. 2-1*D*). This finding was consistent with the finding at FCz (Fig. 2*D*). The PE was not correlated with time–frequency power in stop-locked epoch (Fig. 2-1*E*, *F*), whether the mean power of stop-success and stop-error trial was regressed out or not.

In sum, conflict anticipation as indexed by P(stop) was positively correlated with the power in low-theta band (3–5 Hz) over a fixation-locked interval, go-RT was negatively correlated with the power in delta-theta band (2–8 Hz) over a go-locked interval, and PE was positively correlated with the power in the low-beta band (12–22 Hz) over a stop-locked interval. These correlations were located on the midfrontal area of the scalp (Fig. 2*H–K*). Together, the results showed that P(stop), go-RT, and PE can be distinguished by time–frequency representations within a trial. Moreover, the time–frequency cluster of P(stop) preceded that of go-RT in time, and the powers of P(stop) and go-RT clusters were negatively correlated, suggesting a potential causal link between the neural activities of P(stop) and go-RT.

### Beamforming source reconstruction

**Figure 3.**
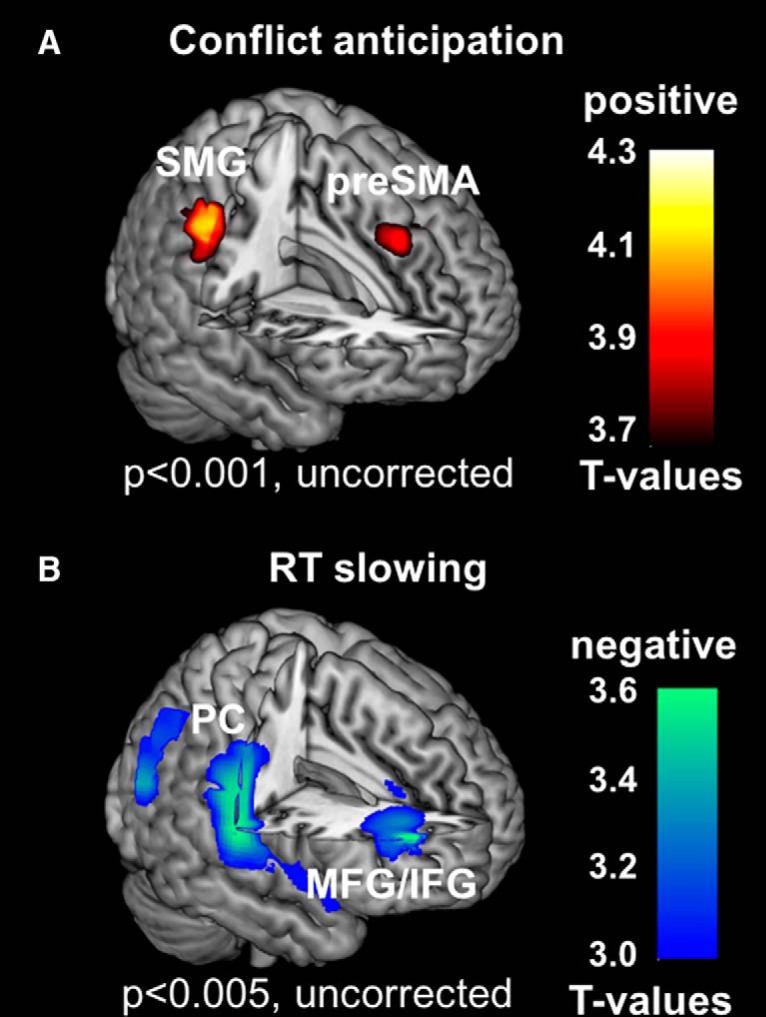
Source reconstruction and localization. EEG correlates of conflict anticipation (*p* < 0.001, uncorrected) were localized to the right SMG and the anterior pre-SMA. ***B***, EEG correlates of RT slowing (*p* < 0.005, uncorrected) were localized to the middle and inferior frontal gyrus (MFG/IFG) and precentral and postcentral gyrus (PC).

Regions reconstructed and localized for the EEG correlates of conflict anticipation and RT slowing are shown in Fig. 3*A* and *B* and summarized in Table 1. At *p* < 0.001 (uncorrected) and cluster size >2000 mm^3^, the time–frequency cluster for P(stop) was localized to the right supramarginal gyrus (SMG) and anterior pre-SMA. These two clusters are significant at *p* < 0.05, corrected for familywise error of multiple comparisons, with small volume correction for the regions of interest as shown in fMRI (Hu et al. 2015a). At the same voxel threshold, the time–frequency cluster for go-RT could not be localized to a significant source. We thus used a more liberal voxel threshold to examine the results. At *p* < 0.005 (uncorrected) and cluster size >20,000 mm^3^, the time–frequency cluster for go-RT was localized to the right precentral and postcentral gyri and middle/inferior frontal gyri. We were unable to reconstruct the source of the power in the low-beta band (12–22 Hz) in association with PE, because there were much fewer (<100) stop trials (in comparison to 350 ± 46 and 257 ± 58 trials that survived artifact rejection and were available for source construction each for conflict anticipation and RT slowing). [Table T2]

**Table 1.** Source reconstruction for the EEG correlates of conflict anticipation (*p* < 0.001, uncorrected; cluster size >2000 mm^3^) and RT slowing (*p* < 0.005, uncorrected; cluster size >20,000 mm^3^)

Cluster size (mm^3^)	*t* value	MNI coordinate (mm)			Side	Identified brain region
		*x*	*y*	*z*
Conflict anticipation						
3,277	4.09	59	–23	50	R	Supramarginal gyrus
2,363	3.86	4	37	42	R/L	Presupplementary motor area
RT slowing						
20,927	3.79	38	61	25	R	Middle and inferior frontal gyri
30,936	3.62	61	–10	7	R	Central operculum, postcentral gyrus

**Table 2. T2:** Statistical table

Location	Data structure	Type of test	Observed power or 95% confidence interval
a	Normal distribution	Paired *t*-test	[36, 53]
b	Normal distribution	Pearson correlation	[0.77, 0.94]
c	Normal distribution	Pearson correlation	[–0.82, –0.97]
d, f, g, j, k, l, m, n, o	Nonparametric	Nonparametric cluster-based permutation test	100%
e	Nonparametric	Nonparametric cluster-based permutation test	98.0%
h	Nonparametric	Wilcoxon signed rank test	[–0.047, –0.002]
i	Nonparametric	Nonparametric cluster-based permutation test	95.5%

## Discussion

We identified time–frequency neural activities associating trial-by-trial conflict anticipation to RT slowing in a stop-signal task. First, we showed that conflict anticipation, quantified by the trial-by-trial likelihood of a stop signal from a dynamic Bayesian model (Yu and Cohen, 2009), was positively correlated with RT slowing across trials, in support of proactive behavioral control. The Bayesian model provides a unique framework to estimate individuals’ expectations from observations and describe how individuals adjust behavior according to these expectations. In EEG, we showed that (1) conflict anticipation was positively correlated with the power at low theta band (3–5 Hz) during the foreperiod but not after go-signal onset; (2) go-trial RT was negatively correlated with the power at delta-theta band (2–8 Hz) after go-signal onset but not during the foreperiod; (3) the power of these two time–frequency clusters was negatively correlated across trials, mirroring the behavioral finding of sequential effect; and (4) stimulus prediction error was positively correlated with the power in the low-beta band (12–22 Hz), after the onset of stop signal. Further, beamforming source reconstruction localized the EEG correlates of conflict anticipation to the supramarginal gyrus and anterior pre-SMA, consistent with previous fMRI studies (Hu et al. 2015a). It is worth mentioning that despite the strong correlation between P(stop) and RT, we were able to identify distinct neural correlates of these behavioral measures by modeling P(stop)- and RT-related activities each at trial and go-signal onsets. This is made possible by the temporal resolution of EEG signals even when the trial and go-signal onsets occurred within 1–3 s. Thus, these findings distinguished the time–frequency electrophysiological correlates of conflict anticipation, response control, and prediction error and characterized the trial-by-trial neural oscillatory dynamics of proactive control of motor response.

The current results showed that theta power reflects the magnitude of both conflict anticipation and RT slowing, and these two activities were correlated in the same trial, broadly in accord with a role of theta oscillations in cognitive control (Cavanagh et al. 2009; Cohen and Ridderinkhof, 2013; Cavanagh and Frank, 2014; Cohen, 2014a, 2016; Chmielewski et al. 2016; van Noordt et al. 2016a, b; Wang et al. 2016). Previous EEG studies of Simon tasks showed that theta power was more positively correlated with RT on high-conflict than on low-conflict trials (Cohen and Cavanagh, 2011; Cohen and Donner, 2013); however, it remains unclear whether theta power primarily reflects conflict processing or behavioral response as a result of the conflict or both. Here, we dissociated the effects of conflict anticipation and response control and showed that theta power correlated with these two processes in opposite directions, and it is possible that the previous findings were mainly driven by conflict anticipation. This new finding suggests that different cortical networks process conflict anticipation and response control, in communication via theta oscillation. These results are also consistent with earlier fMRI findings that distinct prefrontal and frontal cortical structures support conflict anticipation and RT slowing, and activities during conflict anticipation Granger caused activities during RT slowing (Hu et al. 2015a, b; Manza et al. 2016).

A substantial body of work supports a role of theta oscillation in proactive control. In the current findings, the onset time of theta power responses of both conflict anticipation and RT slowing started as early as ∼0–200 ms after stimulus onset, favoring the explanation that these responses reflect preparation for the upcoming event rather than exogenously elicited reactions. An EEG study of conflict tasks showed that RT was better predicted by ongoing endogenous (non–phase-locked) than exogenous (phase-locked) theta power, supporting a top-down, proactive rather than bottom-up, reactive process (Cohen and Donner, 2013). In an anti-saccade task, medial frontal theta power was increased during response preparation on correct but not error trials (van Noordt et al. 2016b). With transcranial alternating current stimulation in the theta frequency applied to midfrontal scalp region, participants slowed down in response during low-conflict trials and as a result exhibited less conflict effect in a Simon task (van Driel et al. 2015). In another EEG study of proactive control, theta oscillation reflected information gathering for proactive control across oddball, go/no-go, and task-switching paradigms (Cooper et al. 2016). More broadly, studies have associated theta oscillations to various top-down cognitive processes to ready attention for task switching (Min and Park, 2010; Daitch et al. 2013; Phillips et al. 2014), maintain working memory (Scheeringa et al. 2009), and encode and retrieve episodic memories (Nyhus and Curran, 2010).

The current findings showed that delta oscillations, in addition to theta oscillations, are correlated with RT slowing. Earlier studies showed delta oscillation in the motor process of cognitive control, in accord with the current findings. For example, delta and theta power followed the motor response during error-related reactive control and in negative association with RT in trial-by-trial correlation (Cohen and van Gaal, 2014). Another intracranial EEG study showed that the phase of delta-theta (2–5 Hz) oscillation modulated high-gamma power (>70 Hz), and a stronger coupling predicted shorter RT in spatial target detection (Szczepanski et al. 2014; Voytek et al. 2015). A recent study of oddball, go/no-go, and switch tasks demonstrated sensitivity of frontal delta and theta power to sensorimotor control (Cooper et al. 2016). In a modified stop-signal paradigm, which manipulated proactive/reactive control (with informative/neutral preparatory cue) in conjunction with selectivity of stopping behavior (unimanual vs. bimanual response), both factors interactively modulated delta power on stop trials (Lavallee et al. 2014). However, it is unclear whether the change of delta power reflects the difference of bottom-up response to stopping cue or top-down conflict anticipation. The current trial-by-trial analyses dissociated these two factors and showed that the delta power was associated with RT but not with conflict anticipation, favoring the explanation that delta power reflects response control rather than signal anticipation.

It has been shown that the theta and delta oscillations were the major frequency-domain features of time-domain N2 and P3 ERP components, respectively (Cavanagh et al. 2012; Huster et al. 2013; Cavanagh and Frank, 2014; Harper et al. 2014; Cavanagh and Shackman, 2015). Evidence is available from response inhibition tasks (e.g. SST, go/no-go) that theta/N2 and delta/P3 each reflects conflict monitoring and motor inhibition (Huster et al. 2013). In particular, the amplitude of anterior N2, generated in medial-frontal cortex, was modulated by the predictability of stimulus (Heinze et al. 1990; Woods et al. 1992), consistent with our finding that theta power reflects conflict anticipation. P3 is associated with response inhibition (Kok et al. 2004; Kenemans, 2015), and the peak latency of P3 is positively correlated with RT (Conroy and Polich, 2007; Polich, 2007). Here, we showed that stronger delta power was associated with shorter RT, and it is possible that the change in delta power is reflected as shift in P3 peak latency in the time domain. However, it is important to note that, as a temporally averaged phase-locked signal, ERP waveform does not distinguish oscillation in various frequency bands, and the non–phase-locked neural oscillations on each trial may cancel out in averaged ERP waveform. The trial-by-trial analysis employed in the current study did not lend itself to revealing potential phase resetting responses, and future study is needed to directly examine this issue.

Beamforming analyses localized the time–frequency correlates of conflict anticipation to the pre-SMA and right SMG and those of RT slowing to right prefrontal and somatomotor cortex, in correspondence to earlier fMRI findings (Hu et al. 2015a). The preSMA is implicated in numerous studies for volitional control of behavior (Rushworth et al. 2004; Kennard et al. 2005; Jaffard et al. 2008; McDowell et al. 2008; Wolpe et al. 2013) and other functions, such as performance monitoring, required for cognitive control, as demonstrated extensively in unit recordings from behaving monkeys (Stuphorn et al. 2000; Ito et al. 2003; Stuphorn and Schall, 2006). Both right-hemispheric prefrontal structures (Li et al. 2008; Cai et al. 2014) and ipsilateral somatomotor cortex (Meyer et al. 1998) are known to be involved in the control of movement initiation. On the other hand, the beamforming results were obtained with an uncorrected statistical threshold. Although the pre-SMA and right SMG were in the locations shown by an earlier fMRI study (Hu et al. 2015a) and significant with small volume correction, the clusters identified for RT slowing were not exactly the same. Thus, while a liberal threshold may be needed to accommodate noisy trial-by-trial EEG responses and a lack of individual MRI-based head model for source reconstruction (Green and McDonald, 2009), these results would require replication.

We also showed that stimulus prediction error was encoded by low-beta band power. This finding is consistent with an earlier report that beta oscillations (∼13–30 Hz) react to unexpected, bottom-up factors (Arnal and Giraud, 2012; Engel and Fries, 2010). In sensory systems, low-beta oscillations (∼13–20 Hz) respond to prediction error, as would occur during an unexpected or oddball stimulus (Arnal et al. 2011; Chang et al. 2016; Fujioka et al. 2009; Haenschel et al. 2000; Kopell et al. 2011). Interestingly, the time–frequency interval of the low-beta response appeared to be similar for auditory prediction error (Chang et al. 2016), potentially suggesting similar neural process across sensory modalities. Another study showed that beta oscillatory synchronization in the frontostriatal network was increased in response to a novel stimulus to support adaptation of ongoing behavior (Wessel et al. 2016). In other studies, the power of high-beta band (∼20–30 Hz) increased to unexpected positive rewards (Cohen et al. 2007; Marco-Pallares et al. 2008) and alpha to beta (∼8–25 Hz) power was negatively correlated with positive prediction error (Cavanagh, 2015). More work is needed to investigate whether low-beta oscillations elicited by stimulus prediction error can be localized to the dorsal anterior cingulate cortex, as shown by fMRI (Ide et al. 2013; Silvetti et al. 2014; Hu et al. 2015a; Manza et al. 2016). Likewise, many studies have shown that alpha oscillations are associated with top-down processes, including sustained alertness, selective attention, and stimulus-driven adaptive control (Sadaghiani and Kleinschmidt, 2016). Given their broad role in cognitive processes, it remains to be seen how alpha oscillations may partake in proactive control in the stop-signal task.

An important consideration is whether or how P(stop) as computed by the Bayesian model relates linearly to the construct of conflict anticipation. For instance, an extreme value of P(stop) (e.g., 0.9) would suggest full anticipation of a stop signal and readiness to stop, thus involving little conflict. On the other hand, P(stop) as estimated by the Bayesian model ranged from 0 to ∼0.45 (see Fig. 2 of Ide et al. 2013 and Fig. 1 of Hu et al. 2015a) with a peak at 0.25—the probability of the stop signal dictated by the experiment. In the current experiment, P(stop) estimates centered around a lower range. Thus, it appears that even at an upper value of, say, 0.4 of P(stop), participants predominantly expect to respond to go signal. If “conflict” reflects the conflicting processes between responding and withholding a response, P(stop) would seem to monotonically reflect the extent of conflict anticipation within this estimated range of values. It is not clear why the P(stop) in the current study spanned a lower range, compared with earlier studies. Of the task parameters, the only difference is a shorter foreperiod in the current EEG (1–3 s) compared to earlier fMRI (1–5 s) studies. This observation suggests the need of more work to investigate the influence of task variables on model outcomes of SST performance.

Another issue pertains to the potential differences in the influence of stop success and error trials on proactive control. A previous study of a saccade countermanding task showed post-success but not post-error slowing in macaque monkeys (Emeric et al. 2007). Although inconsistent with earlier work of choice RT tasks, this finding suggests that monkeys may perform to optimize their reward (water intake) by executing fast saccades most of the time and only slowing down after successfully cancelled (rewarded) trials, in favor of positive versus negative reinforcement. Stop success and error trials may involve distinct psychological processes and contribute to proactive control via distinct mechanisms according to experimental contexts (Li et al. 2008; Chang et al. 2014; unpublished observations). New models would be needed to capture the potential differences in the influence of success and error trials and, more broadly, how positive and negative reward contingencies may modulate cognitive control differently.

In conclusion, the current study provides evidence for distinct time–frequency neural mechanisms of conflict anticipation and behavioral slowing in the stop-signal task. Conflict anticipation was reflected in the power in low-theta band (3–5 Hz) during the foreperiod, and behavioral slowing was reflected in the power in delta-theta band (2–8 Hz) during motor response. The magnitude of power in these time–frequency clusters was significantly correlated on a trial-by-trial basis. These findings substantiate a neural oscillatory mechanism associating conflict anticipation to behavioral adjustment.

## Synthesis

Reviewing Editor: Bradley Postle, University of Wisconsin Decisions are customarily a result of the Reviewing Editor and the peer reviewers coming together and discussing their recommendations until a consensus is reached. When revisions are invited, a fact-based synthesis statement explaining their decision and outlining what is needed to prepare a revision will be listed below. The following reviewer(s) agreed to reveal their identity: Michael Cohen, Patrick Bissett.

As you can see, you received an excellent set of reviews, with one reviewer particularly expert on EEG analyses and the other on the theoretical and empirical study of Stop tasks. Both make recommendations that will require some additional analyses and perhaps some expository modifications, but neither raises serious conceptual concerns, nor suggests the need for additional data collection.

R#1

This study makes a good contribution to the literature. It should definitely be published and I think will be well-received. But before giving the green light, I have some concerns that should be addressed.

Please insert the university in lines 85/6.

Page 9: -500 to 1000 is not a “longer epoch.” I would actually say it's on the short side for time-frequency analyses. This leads to my major concern:

Edge artifacts can last a few cycles per frequency. With only 200 ms between epoch onset and baseline onset, only the highest frequencies analyzed here are truely safe from edge artifacts. All the lower frequencies, including theta, have the risk of contamination. It's possible that the results are OK, but I would not feel comfortable with using such short time windows. There should be at least a full cycle of the lowest frequency on either side of the time window of interest, and preferably closer to three. Or maybe Besa does something to account for this? If so, the procedure must be described. If not, the authors should re-analyze the data using larger epochs (the “buffer zones” could be excluded from the final results and therefore plots and statistical evaluations).

Line 182: Wavelet convolution undeniably extracts time-frequency features from the data. Whether those features are “neural oscillations” is a whole other can of worms (this is discussed in many places, including the Cohen and Donner 2013 paper cited by the authors). I doubt the authors want to get caught up in this (rather obtuse and, at times, semantic) issue. Therefore, I recommend softening the language to speak more concretely about time-frequency patterns/results/responses, as leave the “hot” term oscillation to the introduction and Discussion sections (i.e., interpretation of the results as opposed to description of the results).

Here is my major conceptual concern: What is p(stop) really telling us? The Bayesian model is not a biophysical model, so it is telling us something conceptual/abstract. The model output correlates extraordinarily strongly with RT (~80% shared variance!). Is p(stop) just a proxy measure for RT? Apparently not, because the authors find dissociations in patterns of time-frequency correlations with p(stop) vs. goRT. I guess my “concern” here is really confusion -- how can two measures that correlate so strongly with each other correlate so differently with the EEG signal? This needs a more in-depth discussion, perhaps also with additional visualizations.

It would be interesting to see the trial series plot of p(stop). At present we see only distributions, but it would be interesting to see how p(stop) changes over trials, e.g., how much temporal variability there is.

line 283/4: what does [a/b] mean in, e.g., 10^(-8 b)?

I don't completely understand the model-fitting procedure. What was the k-order (trial history) of the model? And what was the balance between the order and the estimates of the coefficients? Maybe it would also help to make a plot of the k's for all subjects.

I have a new policy that I never recommend acceptance of a paper until analysis code is put online (personal website, github, google-code, figshare, supplemental materials, anything), with a clear description and link in the paper. Preferably the data should be posted as well, but at least the analysis and Bayesian modeling code.

R#2

In this work, the authors are interested in conflict and proactive control. To these ends, they relate behavior in the stop-signal task to electrophysiology. They show a few related results. First, they show that the probability of a stop signal was positively correlated with power in the low theta band at fixation. Second, they show that go RT was negative correlated with power in the wider delta-theta band at go stimulus onset. Third, they show that stimulus prediction error was positively correlated with power in the low-beta band at stop signal onset. Finally, they show that power of the probability of stop cluster and go-RT cluster are negatively correlated, suggesting that anticipating a stop signal is related to slower RT.

The authors explore a fundamental and importantly set of constructs, specifically, the relationship between proactive control, conflict, and response control. For this reason, I am enthusiastic about the paper. However, the following set of issues somewhat dampen my enthusiasm.

1. The authors seem to neglect a large swath of the stopping literature that has addressed sequential control adjustments. This includes (but it not limited to) the following work:

Bissett & Logan, 2011 JEP:LMC

Morein-Zamir et al., 2007 JEP:HPP

Rieger & Gauggel, 1999 British J of Psych

Verbruggen et al., 2008 JEP:HPP

Verbruggen & Logan, 2008 JEP:HPP

Verbruggen & Logan, 2008 QJEP

Verbruggen & Logan, 2009 JEP: HPP

Points 2 and 3 below also relate to this issue. Much of this previous work is focused on the same theoretical space of the current manuscript, including conflict, expectancy, and proactive vs. reactive control, and it uses the same basic paradigm (stop signal). Much of this work also presents alternative, simpler methods for computing sequential control adjustments (e.g., compare RT after a stop signal to RT before a stop signal). In order to maximize the impact of this work, it seems necessary to more thoroughly synthesize the current ideas with the existing literature.

2. The authors cite Lavallee et al., 2014 in passing, an EEG study of the time-frequency characteristic of stopping behavior. Considering the overlap in methods and goals and the cursory mention of it in the text of this manuscript, the reader is left to wonder how this current work goes beyond that previous work. I recommend the authors draw a clearer distinction between what has been done in previous work and what is new here.

3. The authors assert that “the extent of conflict adaptation --[is the] belief of an upcoming stop signal...”. However, the relationship between p(stop) and the concept of conflict remains unclear. Is it a linear, increasing function such that higher p(stop) yields higher conflict adaptation? What if the p(stop) approaches 1? Is it maximal at 50% p(stop) because there is maximal uncertainty about the upcoming behavior (i.e., go or stop)?

On a related point, others, including Schall and colleagues (e.g., Emeric et al., 2007 Vision Research; Ito et al., 2003 Science; Stuphorn & Schall, 2006 Nature Neuro; Stuphorn et al., 2000 Nature) have suggested that conflict is maximal specifically on successful stop (not failed stop or the anticipation of either stop). Can the current instantiation of conflict and conflict adaptation be synthesized with the way that Schall and colleagues discuss conflict?

4. Though there is some historical precedent for a simple go task (e.g., Lappin & Eriksen, 1966 JEP; Logan, Cowan, & Davis, 1984 JEP:HPP; Ollman, 1973 A&P IV), the overwhelming majority of stop-signal tasks use a choice go task. Here, the RTs (405ms) are much longer than are normally observed in a simple RT tasks, both without (Donders 1868/1969 Acta Psychologica) and with (Logan, Cowan, & Davis, 1984 JEP:HPP) stop signals. Therefore, it appears that subjects are not taking the instructions to respond quickly to the go signal very seriously.

In order to ensure that the combination of the somewhat unusual simple go task and the considerable go RT slowing does not affect any of the main dependent variables, I recommend some follow-up analyses/questions. Does RT slow over the course of the experiment (if so, SSRT may need to be adjusted: Verbruggen, Chambers, & Logan, 2013 Psych Science)? Is there considerable individual differences in this slowing? Is the race model violated in any subjects (i.e., are RT as long or longer on stop-failure trials than go trials?)? Did a subset of subjects wait until the RT deadline of 1s? Was there any sign that the upper tail of the RT distribution was truncated for any subjects by the 1s deadline? Did the SSD tracking algorithm “work” (i.e., stop success rate ~50%) for all subjects? It seems that the addition of analyses like the ones that I just mentioned could help the reader to ensure that the performance is not aberrant in any way.

5. Related to point #4, the authors note that many trials (24.1%) were removed in preprocessing. The authors say that this might have occurred because of long epochs. Were there more exclusion later in the epochs, which would be consistent with this idea? Also, did the trials that were excluded differ from the trials that were included in behavior? With such a large rate of exclusion, it seems necessary to ensure that these 24.1% of trials with artifacts in their EEG signal did not also include anomalous behavior that perhaps should be understood instead of thrown out.

Smaller issues:

Why use a maximum likelihood procedure to find the critical SSD? What does this buy over more standard methods like taking the mean/median SSD?

It was unclear why P(stop) in figure 1 was on average so low. It may be worth unpacking why it isn't .25 (like the overall probability of a stop trial in the experiment).

The thresholds for voxel clustering (2,000mm3 and 20,000mm3) were seemingly not justified by any means, citations or otherwise. Such a choice seems to require some form of justification.

The authors focus on a single electrode, and I understand the desire to reduce multiple comparisons. However, it would seem useful to at least investigate (in a separate, post-hoc analyses) the possibility of additional activation across other electrodes to see if any useful discoveries could be made through exploration.
