# Supplementary figures and images for: Proactive Control: Neural Oscillatory Correlates of Conflict Anticipation and Response Slowing
Source: eNeuro. 2017 May 26;4(3):ENEURO.0061-17.2017. doi: 10.1523/ENEURO.0061-17.2017 (PMC5446487; doi:10.1523/ENEURO.0061-17.2017)

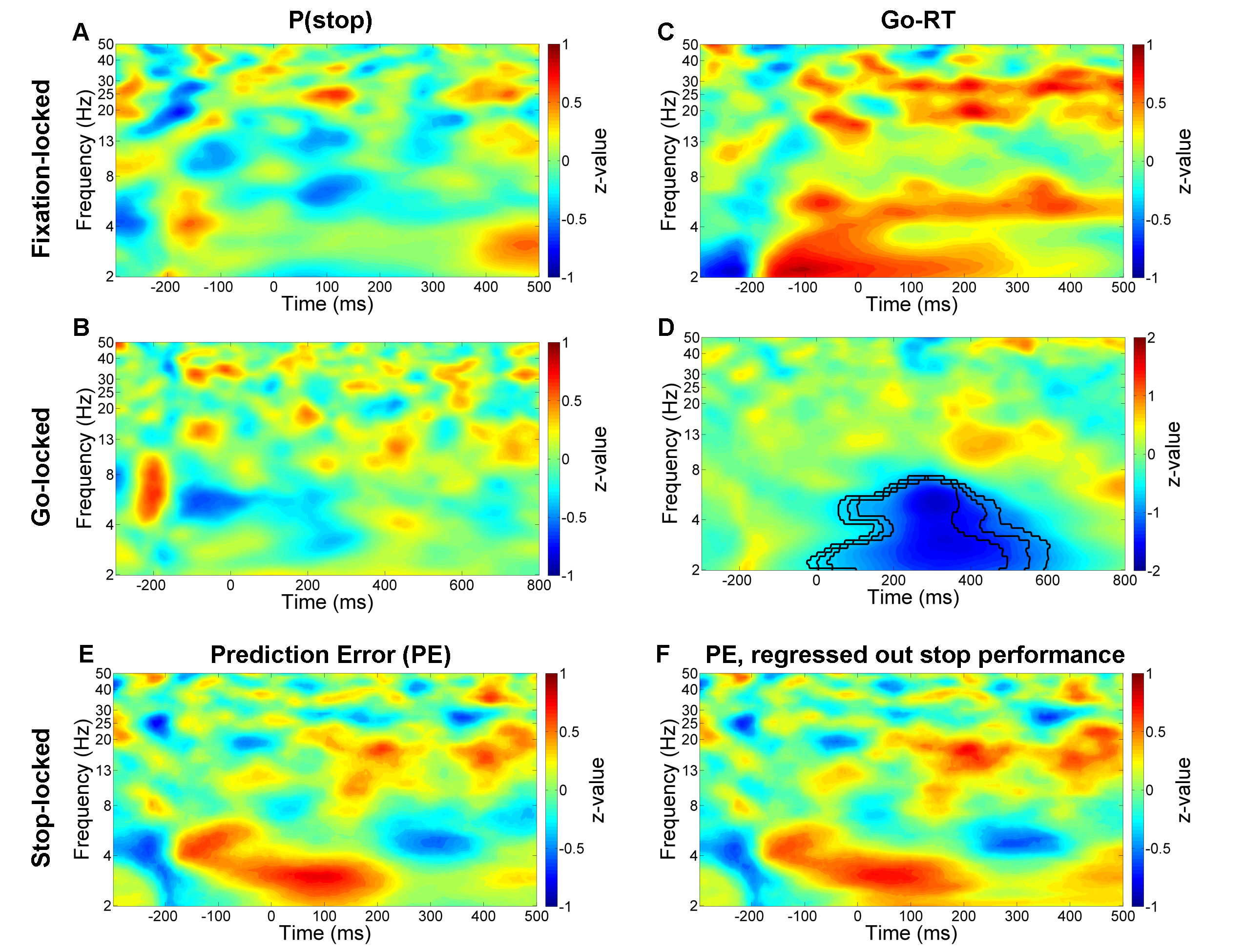

Supplement: Figure 2-1 — Trial-by-trial oscillatory power correlates of P(stop), go-RT, and PE at channel Pz. The format is the same as with Fig. 2. The color represents the z-value of Spearman correlation, and the black contours represent statistically significant time–frequency clusters in the nonparametric cluster-based permutation test across participants, with clustering threshold at p < 0.02, 0.005, and 0.001 levels (see Materials and Methods for details). A, B, Time–frequency power was not correlated with P(stop) in fixation-locked or go-locked epoch. C, Time–frequency power was not correlated with go-RT in fixation-locked epoch. D, Time–frequency power was negatively correlated with go-RT in the intervals 2–8 Hz and ∼100–500 ms in go-locked epoch. E, F, Time–frequency power was not correlated with PE in stop-locked epoch.. Download Figure 2-1, TIF file. [file enu003172319so4.tif]
